# A scalable hybrid computational intelligence framework with bio inspired optimization for high dimensional malicious URL inference

**DOI:** 10.1038/s41598-026-44851-4

**Published:** 2026-03-24

**Authors:** Hua Liu

**Affiliations:** College of Electronic and Information Engineering, Jiuquan Vocational Technical University, Jiuquan, 735000 Gansu China

**Keywords:** Computational intelligence, high-dimensional inference, bio-inspired optimization, internet-scale analytics, malicious URL detection, discriminative modeling, feature attribution, network-layer analysis, Engineering, Mathematics and computing

## Abstract

The increasing complexity and scale of internet infrastructure demand computational frameworks capable of performing accurate, scalable, and interpretable inference over high-dimensional network data. Conventional detection strategies often struggle to maintain robustness and efficiency when confronted with heterogeneous feature spaces and rapidly evolving threat patterns. This study presents a scalable hybrid computational intelligence framework that integrates discriminative statistical modeling, gradient-based inference, and bio-inspired meta-heuristic optimization to address large-scale malicious URL detection. The proposed framework couples Linear and Quadratic Discriminant Analysis with a categorical gradient-boosted inference engine, while automated parameter exploration is conducted using the Mother Optimization Algorithm and the Osprey Optimization Algorithm. A large-scale dataset consisting of 63,191 URLs, described by both application-layer and network-layer attributes, is employed to rigorously evaluate the framework’s performance. Statistical robustness is evaluated through exploratory distribution assessment (Shapiro–Wilk), nonparametric hypothesis testing (Kruskal–Wallis), pairwise model comparison, and cross-validation-based performance consistency. These procedures provide quantitative support for model comparison and feature relevance under non-Gaussian conditions. Model transparency and reproducibility are further strengthened using SHAP-based feature attribution to quantify the influence of individual variables. Results demonstrate that the bio-inspired optimized models achieves superior performance, attaining an accuracy of 96.35%, precision of 96.54%, recall of 96.35%, F1-score of 96.40%, and specificity of 96.36%. These findings indicate that the synergistic integration of hybrid discriminative intelligence and bio-inspired optimization significantly enhances inference capability in real-world URL classification dataset with moderate-dimensional features. Beyond cybersecurity, the proposed framework offers a transferable and computationally efficient paradigm for high-dimensional classification and decision-making tasks across engineering systems and data-intensive scientific applications.

## Introduction

### Study background

The internet is a vital resource for communication, commerce, and the exchange of information in our digital era. However, despite its benefits, the internet still faces some serious security issues related to cybercrime, among which rogue websites hold a prominent position^1,2^. These websites are packed with malware and phishing schemes, which are simply viruses, to get a victim’s personal data or to ruin their device. Heuristic methods, URL-based feature extraction, and URL-based blacklisting are some of the conventional ways used for the detection of harmful web resources^3,4^. Nevertheless, the precision of these methods in terms of completely identifying and preventing access to different types of malicious websites is minimal. URL blacklisting is the process of recording URLs known to be harmful and blocking access to websites that contain them^5,6^. In part, this method can achieve success, but it fails to handle the newly created rogue sites and those that generate URLs dynamically. Additionally, the use of URL obfuscation methods or frequent URL changes makes it easy for thieves to bypass detection^7^.

Heuristic techniques, in an attempt to identify malicious URLs based on properties such as URLs, HTML content, or behavior, rely on fixed rules or patterns^8^. While heuristic methods may catch some malicious activities, they usually generate a large number of false positives and may overlook sophisticated threats that do not match known patterns^9,10^. Feature extraction from the URL breaks down the URL into its elements and gathers information such as domain repute, URL length, and whether certain keywords exist. Although this method may reveal a website’s basic features, it cannot tell the whole story or convey the semantics of the data on the page^11^. Despite all these efforts, the continuous, unpredictable changes in malicious websites beyond the capabilities of traditional detection methods continue to occur^12^. To escape the current blacklist, heuristic, and feature extraction methods, attackers regularly employ obfuscation, dynamic URL generation, and content mimicry^13,14^. Consequently, there is a strong need for more advanced, adaptive, and intelligent frameworks that employ machine learning algorithms, optimization strategies, and statistical validation to develop solutions that are dependable and scalable^15^. This study is motivated by the aforementioned shortcomings and aims to contribute by facilitating the establishment of a detection framework that not only improves accuracy, intervention possibilities, and security against complex threats but also makes it available and dependable^16^.

### Related work

Madhusudhan et al.^17^ concentrated on the problem of finding bad web pages that keep being created using methods that make the pages look smarter. Besides, they defined the limitations of blacklist-based methods and the only way content-based feature extraction was done, i.e., by physically visiting the site, which introduced the danger of malicious activity. They employed NLP and TF-IDF methods to create a collection of terms that are both new and common to benign and malicious URLs. Next, they applied an Artificial Neural Network (ANN) for classification, and the performance was benchmarked with other machine learning techniques. The results demonstrated that the ANN achieved the highest accuracy of 96.70%, thereby demonstrating the promise of using NLP-facilitated feature extraction alongside deep learning methods for detecting malicious websites.

Hussain et al.^18^ explored the limitations of current methods for identifying malicious websites, highlighting their slow, inconsistent, and difficult-to-compare nature across studies. To resolve such issues, the authors introduced a comprehensive framework using the XGBoost classifier and a Kaggle dataset for model development and validation. Their process involved data collection, data cleaning, and classification, followed by a detailed performance evaluation. The aim was to enhance the precision and trustworthiness of detecting malicious websites using the new method compared to traditional methods. The XGBoost-based method they proposed achieved a precision of 95.5%, surpassing several existing methods. Their research not only demonstrated the effectiveness of the combination-based machine learning technique but also released a framework open to others for developing detection systems for malicious websites with higher accuracy and consistency. Liaquathali and Kadirvelu^19^ explored the limitations of traditional signature-based methods in detecting malicious webpages, emphasizing the need for proactive approaches that can adapt to rapidly evolving cyber threats. Their study proposed a content-based detection framework that leverages a pretrained Word2Vec model combined with multiple machine learning classifiers. The webpage content was first encoded into Word2Vec embeddings, and the average embeddings were used as features for classifier training. Among the tested models, Random Forest and Extreme Gradient Boosting (XGBoost) achieved the strongest performance, with the Random Forest classifier recording an accuracy of 94.8% and an F1-score of 94.9%, while XGBoost achieved an accuracy of 94.6% and an F1-score of 94.7%. Their findings demonstrated that Word2Vec-enhanced feature representations substantially improve malicious webpage detection, highlighting the importance of integrating semantic content analysis into cybersecurity frameworks.

In addition, Recent work in malicious URL detection has explored transformer-based and deep learning approaches. For instance, transformer models such as BERT, GPT-3.5, and RoBERTa have been used to generate contextualized features from URLs and web content, achieving competitive performance in phishing and malware classification tasks. One study utilizing RoBERTa-Large with metadata attention reported approximately 98% accuracy, outperforming traditional ML models while providing explainable feature contributions^20^. Another line of research focuses on transformer encoders specifically tailored for URL data, such as urlBERT, which learns URL representations for use in classification or multi-task settings^21^. Additional architectures, such as TransURL and semantic-aware BERT+LSTM hybrids, have further demonstrated advantages in capturing both local and global URL patterns^22^.

These transformer-based systems typically rely on deep contextual embeddings and attention mechanisms to model the sequential and semantic aspects of URLs, whereas the presented hybrid approach integrates statistical models and bio-inspired optimization to achieve efficiency, interpretability, and scalable learning over structured feature sets.

### Study objective

Although current machine learning methods have achieved success in detecting malicious websites, their performance is still not optimal, and numerous blocks remain. Existing studies primarily employ content-based or blacklist methods, which lack sufficient capacity to handle dynamically generated URLs, obfuscated links, and hackers’ evolving strategies. Besides, while the performance of ML methods such as ANNs, XGBoost, Logistic Regression, and Word2Vec-based classifiers may be very encouraging, they are not always successful in fully exploiting hybrid modeling and bio-inspired optimization techniques to improve predictive accuracy and interpretability. To overcome these defects, the present study introduces a unique hybrid setting which combines Quadratic Discriminant Analysis (QDA), Linear Discriminant Analysis (LDA), and CatBoost (CAT) with bio-inspired optimizers, i.e., the Mother Optimization Algorithm (MOA) and the Osprey Optimization Algorithm (OOA). The framework is designed to categorize websites as either harmful or safe by utilizing a dataset comprising 1781 URLs with both application-layer and network-layer features. Methods of statistical validation, such as the Kruskal–Wallis test, Tukey HSD, Shapiro–Wilk test, and SHAP analysis, are employed not only to evaluate the model’s functioning but also to identify the most important features. The novelty of this study lies in integrating discriminant analysis, boosting methods, and bio-inspired optimization, yielding hybrid models that achieve significantly better performance than single classifiers. The strategy not only yields higher detection rates but also provides human-readable, resilient problem-solving methods in cybersecurity, thereby constituting a versatile research framework for future detection of malicious websites.

Although individual components such as LDA, QDA, CatBoost, and metaheuristic optimization have been explored independently in prior cybersecurity studies, the present study is not intended as simple algorithm stacking. The proposed architecture is motivated by complementary inductive biases: LDA captures global linear separability, QDA models class-specific covariance structures enabling nonlinear discrimination, and CatBoost learns higher-order feature interactions while natively handling heterogeneous attributes. This structured integration explicitly addresses bias–variance trade-offs and geometric complexity in high-dimensional URL feature spaces. Unlike conventional single-model pipelines optimized in isolation, the framework jointly leverages statistical discrimination and gradient-based ensemble inference, forming a unified hybrid system optimized at the architectural level.

### Contributions

The main contributions of this study are summarized as follows:

- A scalable hybrid computational intelligence framework is proposed that integrates Linear Discriminant Analysis, Quadratic Discriminant Analysis, and CatBoost for high-dimensional malicious URL inference, enabling complementary linear, nonlinear, and ensemble-based discrimination within a unified architecture.
- Bio-inspired optimization using the MOA and OOA is incorporated to jointly tune model components, improving predictive performance while maintaining lightweight inference complexity.
- A statistically grounded evaluation pipeline is established using exploratory distribution analysis and nonparametric hypothesis testing to identify discriminative features under non-Gaussian conditions, complemented by SHAP-based explainability for transparent feature attribution.
- Comprehensive experimental validation is conducted on real-world URL data using stratified 5-fold cross-validation, demonstrating consistent improvements in accuracy, precision, recall, F1-score, and specificity over baseline models.
- Computational cost and scalability are explicitly quantified through runtime and memory analysis, showing that optimization overhead is confined to training while inference remains efficient, supporting practical deployment.
- Reproducibility is enhanced by reporting hyperparameters, optimization settings, cross-validation strategy, and hardware specifications, enabling independent replication of the proposed framework.

## Materials and methods

### Data description and statistical analysis of features

According to Urcuqui et al.^23^, the research’s data came from a set of both normal and dangerous websites that were assembled as part of a web security study. There were 1781 records in the original dataset (216 malicious and 1565 benign URLs). No subsampling or dataset reduction was applied. After preprocessing, including duplicate removal and handling of missing values, the complete dataset was retained to avoid introducing sampling bias. The class distribution was preserved in its original form, ensuring representative coverage of both benign and malicious URLs. This strategy enhances reproducibility and supports the external validity of the reported results. This results in a sample-to-feature ratio greater than 350:1, substantially exceeding commonly recommended thresholds for stable supervised learning. Although the dataset does not represent internet-scale traffic in terms of volume, it reflects realistic URL characteristics commonly used in phishing and malicious website detection research. Therefore, conclusions are restricted to datasets of comparable size and feature dimensionality.

**Fig. 1:**
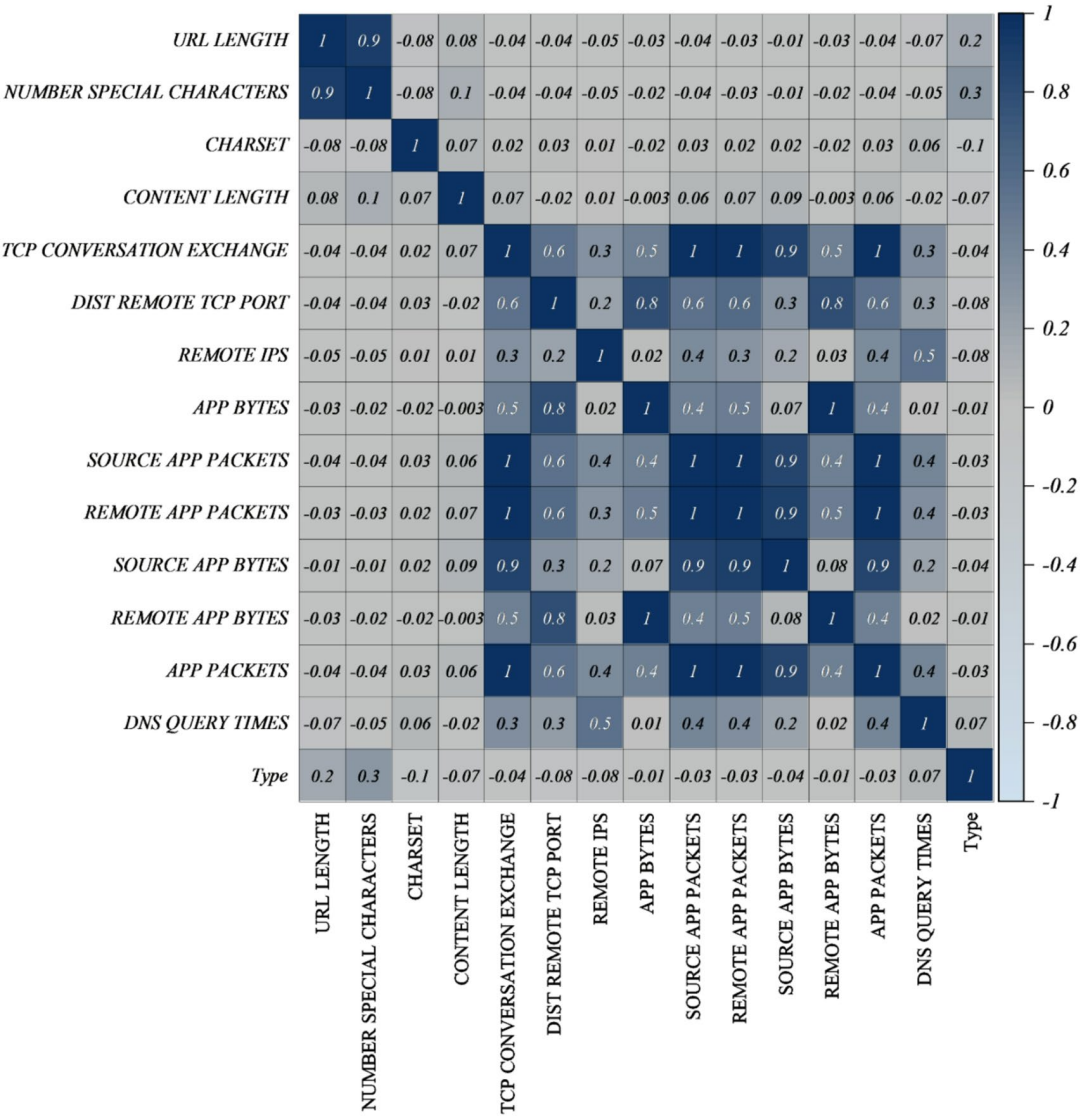
Correlation plot showing the relationships between features in the dataset.

Each URL was examined using Python scripts in order to obtain the network and application layer features. These included WHOIS-based attributes (WHOIS Country, WHOIS State pro, WHOIS Reg date, and WHOIS Updated Date), server-related metadata (Server, Charset, and Content Length), and lexical properties (e.g., URL Length, Number Special Characters) at the application layer. Through the use of a low-interaction honeypot, network-layer features such as DNS query time, App Packets, Source App Packets, App Bytes, Remote App Bytes, App Packets, Dist Remote TCP Port, TCP Conversation Exchange, and Remote IPs were acquired. The target variable TYPE signifies if a website is benign (0) or malicious (1). An analysis of Pearson correlation was conducted in order to gain a better understanding of the relationships between the variables. Figure 1 illustrates the relationship between features, where higher positive correlations are represented by more intense blue shades, and higher negative correlations are represented by more intense brown shades. According to the study, there is a strong correlation between a number of network features, including App Bytes, Source App Packets, and Remote App Packets. However, there have been changes in the number of correlations between specific application-layer attributes.

**Fig. 2:**
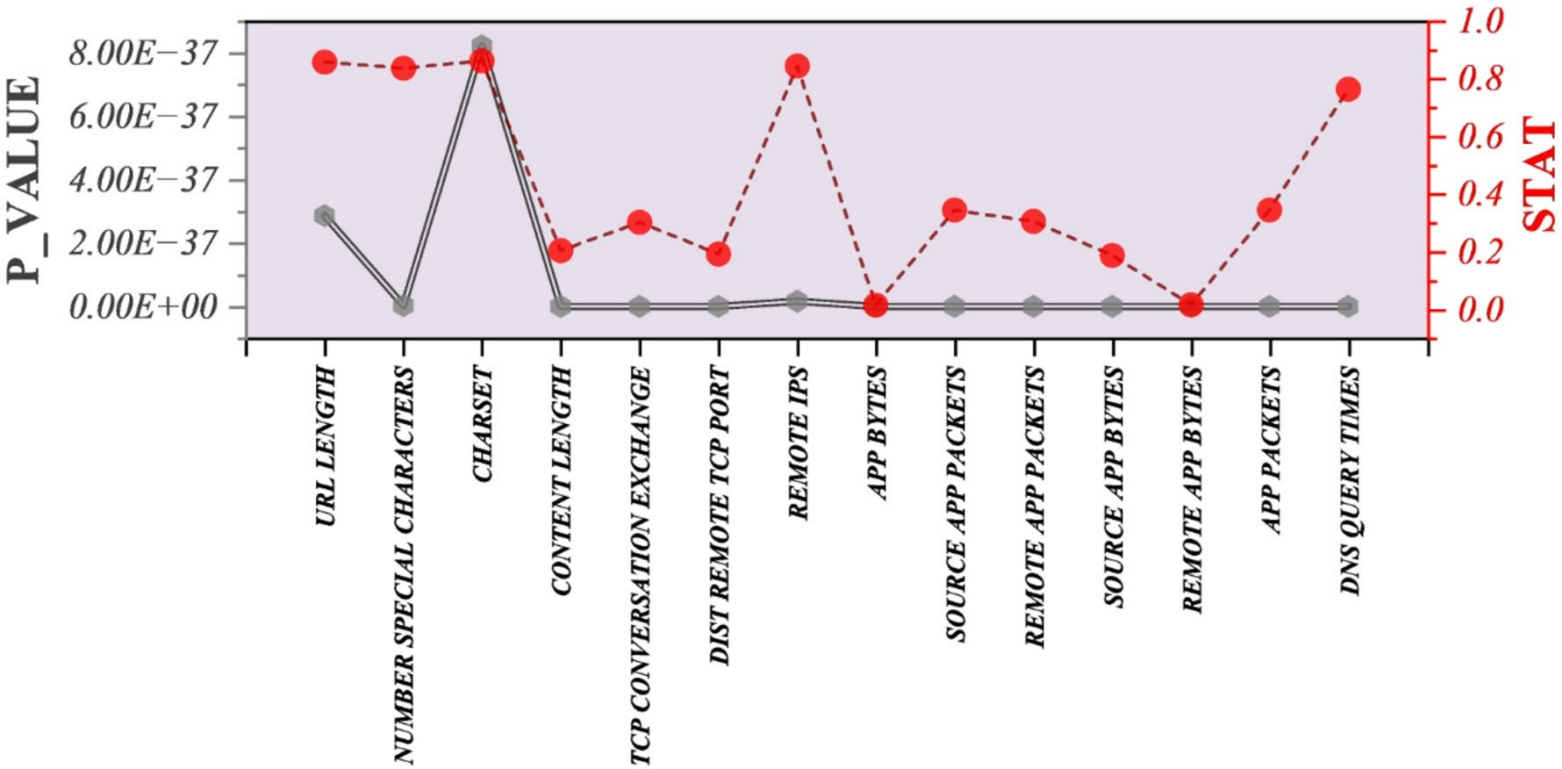
The Shapiro–Wilk test assesses the normality of the dataset.

The Shapiro–Wilk test (Fig. 2) was performed to evaluate the normality of the input variables. For all features, the test yielded *p*-values far below 0.05, indicating significant deviations from normality. For example, URL Length (*W* = 0.8588, *p* = 2.87 × 10^–37^), Number Special Characters (*W* = 0.8385, *p* = 3.46 × 10^–39^), and *DNS_QUERY_TIMES* (*W* = 0.7645, *p* = 8.41 × 10^–45^) all strongly rejected the null hypothesis of normal distribution. The same trend was observed across all other variables, with *W* values substantially below 1.0 and *p* ≈ 0.000. This outcome indicates that the dataset is non-normally distributed, a common property in cybersecurity data where features such as packet exchanges, remote port distributions, or content length often display skewness and heavy tails due to the irregular nature of malicious activity compared to benign traffic. Although the Shapiro–Wilk results confirm non-normality in the predictors, the one-way ANOVA and subsequent Tukey HSD comparison between benign (Type = 0) and malicious (Type = 1) websites still identified statistically significant differences. ANOVA is generally robust to violations of normality when the sample size is large (here, *n* = 1779 residual degrees of freedom), which helps justify its application. The significant finding that malicious websites have substantially lower mean values than benign ones should be interpreted in the context of this non-normality, acknowledging that distributional irregularities are inherent to real-world malicious web data.

**Fig. 3:**
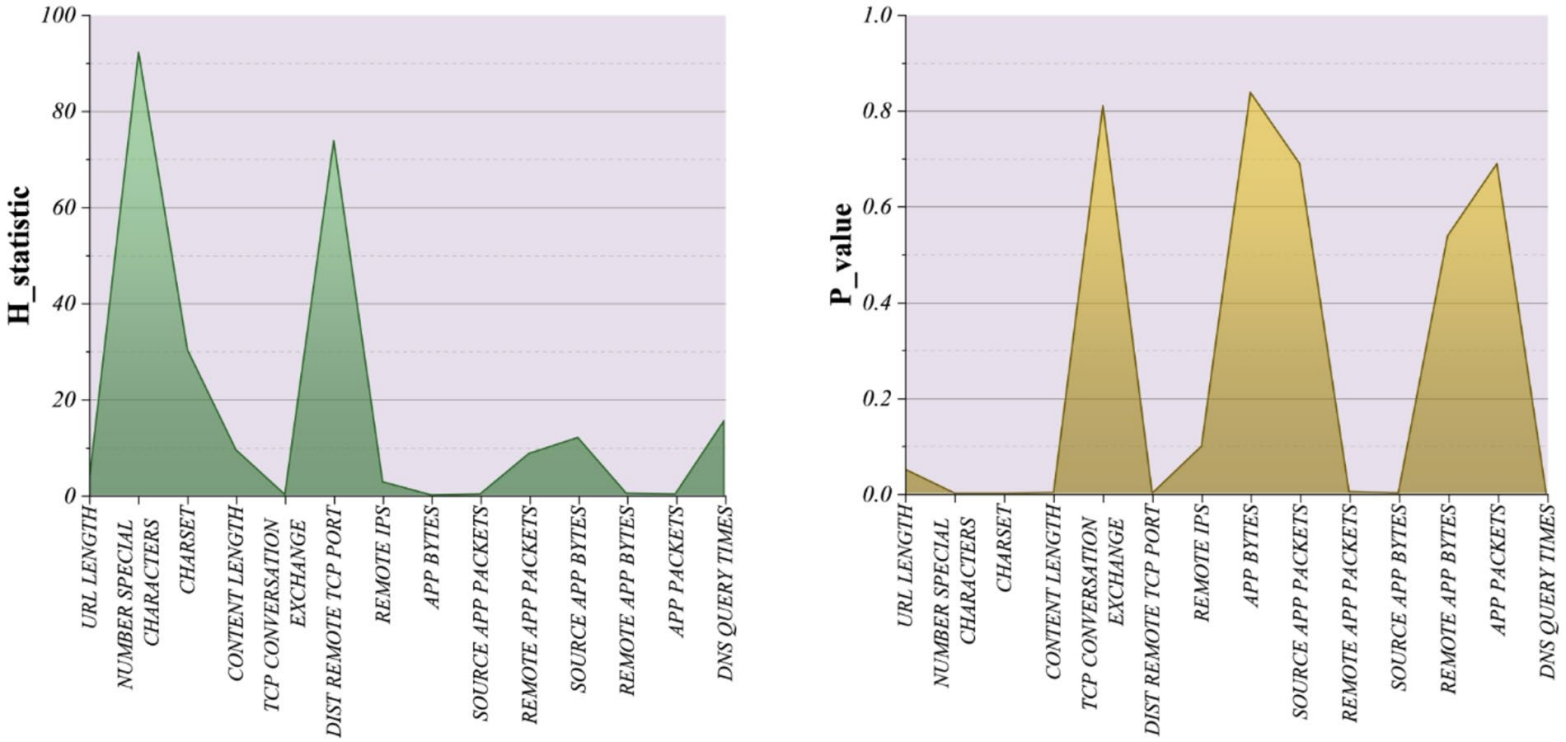
Kruskal-based feature selection highlights the most significant variables contributing to the model.

Given the non-normal distribution of the input variables (as confirmed by the Shapiro–Wilk test), the Kruskal–Wallis test was applied to assess whether significant differences exist between benign (Type = 0) and malicious (Type = 1) websites for each feature. This non-parametric approach is robust to distributional irregularities and provides a more reliable criterion for feature selection in this context. The results (Fig. 3) show that several features demonstrated statistically significant differences between the two groups (*p* < 0.05). Notably, Number of Special Characters (*H* = 92.29, *p* = 7.49 × 10^–22^), Distal Remote TCP Port (*H* = 73.89, *p* = 8.27 × 10^–18^), Charset (*H* = 30.28, *p* = 3.74 × 10^–8^), and DNS Query Times (*H* = 15.43, *p* = 8.57 × 10^–5^) emerged as highly discriminative variables. Other features, such as Content Length (*H* = 9.44, *p* = 0.0021), Remote App Packets (*H* = 8.65, *p* = 0.0033), and Source App Bytes (*H* = 11.94, *p* = 0.0005), were also significant. In contrast, variables such as TCP Conversation Exchange, App Bytes, Source App Packets, App Packets, and Remote App Bytes yielded non-significant results (*p* > 0.05), suggesting limited discriminative power for differentiating between malicious and benign websites.

The Kruskal–Wallis findings are consistent with the Tukey HSD post-hoc results, which showed systematic differences between malicious and benign web types. By identifying the most significant features, the Kruskal–Wallis test provides a data-driven basis for feature selection, ensuring that subsequent classification models focus on attributes that truly differentiate malicious from benign websites. This enhances both the interpretability and predictive accuracy of the malicious website detection framework.

Since multiple features were evaluated simultaneously, applying independent statistical tests can lead to inflated false-positive rates. To address this issue, Kruskal–Wallis p-values were adjusted using the Benjamini–Hochberg false discovery rate (FDR) procedure. This correction controls the expected proportion of false discoveries while preserving statistical power. Table 1 presents the ranked features based on BH-adjusted p-values. Only five features satisfy the FDR significance criterion (FDR < 0.05): NUMBER_SPECIAL_CHARACTERS, URL_LENGTH, CHARSET, DNS_QUERY_TIMES, and REMOTE_IPS. These features were therefore retained for subsequent model training, while the remaining variables were excluded because they were not statistically significant after correction. This ranked, FDR-controlled selection replaces the earlier figure-based presentation and provides transparent quantitative justification for feature inclusion.

**Table 1:** Ranked features after Benjamini–Hochberg correction.

Feature	ANOVA (p_value)	BH_adjusted (p_value)	Significant (FDR < 0.05)	Rank
NUMBER_SPECIAL_CHARACTERS	1.19E-33	1.66E-32	TRUE	1
URL_LENGTH	5.91E-12	4.14E-11	TRUE	2
CHARSET	1.50E-07	7.02E-07	TRUE	3
DNS_QUERY_TIMES	4.60E-04	1.61E-03	TRUE	4
REMOTE_IPS	8.76E-04	2.45E-03	TRUE	5
CONTENT_LENGTH	5.27E-02	6.63E-02	FALSE	6
DIST_REMOTE_TCP_PORT	8.36E-02	9.72E-02	FALSE	7
SOURCE_APP_BYTES	6.43E-02	1.12E-01	FALSE	8
TCP_CONVERSATION_EXCHANGE	8.99E-02	1.40E-01	FALSE	9
SOURCE_APP_PACKETS	1.47E-01	1.87E-01	FALSE	10
APP_PACKETS	1.47E-01	1.87E-01	FALSE	11
REMOTE_APP_PACKETS	1.65E-01	1.93E-01	FALSE	12
APP_BYTES	6.35E-01	6.43E-01	FALSE	13
REMOTE_APP_BYTES	6.43E-01	6.43E-01	FALSE	14

### Categorical boosting (CatBoost)

CatBoost is a sophisticated gradient boosting algorithm designed to handle categorical data while minimizing overfitting and bias efficiently. One of the main reasons for the success of CatBoost as a high-performance ensemble method is that it essentially constructs the following tree(s) based on the residual errors of the previous ones. With such an iterative algorithm, they are able to select the complex, non-linear relationship between features and target variables, which ultimately leads to better performance for classification than in the case of conventional tree-based methods^24^. CatBoost is an effective model for identifying malicious websites, as it combines high-dimensional and heterogeneous features into a single predictive framework. It employs the method known as “ordered boosting” to reduce prediction bias and prevent target leakage. This feature is especially relevant in cybersecurity areas, where datasets often contain a mix of numerical and categorical indicators. The excellent generalization of CatBoost allows it to adjust to changes in malware URLs; thus, it can be used in security applications that are close to the real world. Its computational efficiency and resistance to overfitting make it suitable for large datasets. CatBoost serves as a baseline boosting model and plays a central role in the hybrid framework^25^. When combined with bio-inspired optimization algorithms, its hyperparameters are fine-tuned for enhanced accuracy, precision, and stability. Its integration with discriminant analysis models adds interpretability and resilience against adversarial patterns. Overall, CatBoost is a valuable tool for detecting malicious websites in various domains.

### Linear discriminant analysis (LDA)

LDA was traditionally a supervised learning method, generally used in classification problems. LDA’s primary purpose is to represent the original data with fewer dimensions in a manner that maximizes the separation of classes. LDA does not rely solely on pure dimensionality reduction techniques, but instead exploits the class labels to conduct the projection process, thereby ensuring that the transformed features highlight the distinctions between the categories. LDA accomplishes this by examining two extremes of variation: the variance within each group and the variance between the means of the groups. By identifying a linear combination of features that maximizes the proportion of between-class variance to within-class variance, LDA allows points from different classes to be located at maximum distances in the new feature space^26,27^. Simultaneously, it reduces the dispersion of points within the same class, thereby increasing the ability to distinguish between classes.

In real-life scenarios, LDA is effective for both binary and multiclass classification problems. It is more applicable in situations where the covariance structures of different classes are approximately similar and the data is normally distributed. Provided the assumption holds, the method is capable of drawing linear decision boundaries that effectively separate different classes. In reference to the detection of malicious websites, LDA serves as a simple yet effective baseline model by transforming sets of complex features, such as URL characteristics and network-based attributes, into more concise representations that reveal the patterns specific to benign or malicious websites. By reducing the dimension of the feature space and consequently strengthening class separability, LDA not only helps lower computational complexity but also makes the process more interpretable. Therefore, it can be considered a valuable member in hybrid systems, where its capabilities can be utilized as support for more advanced ensemble and optimization-based classifiers.

### Quadratic discriminant analysis (QDA)

QDA is a supervised classification method that extends the concept of LDA to allow each class to have its own covariance matrix. This difference enables the QDA to model complex, non-linear separations between classes, making it especially suitable when class distributions are significantly different. In contrast to LDA, which assumes a common covariance matrix for all classes and provides linear separation, QDA relaxes the constraint and offers quadratic decision boundaries that may display more explicit geometrical features of the dataset^28^. The advantage of QDA lies primarily in its ability to flexibly handle the presence of a wide range of variability within each class. The same is true for the field of cybersecurity, especially for the problem of malicious website detection, where adaptability can be turned into a resource of strength. Malicious and benign websites differ significantly and may exhibit asymmetric patterns in their lexical, structural, and network-layer features. As variability is taken into account, QDA can easily separate two classes, sometimes even when the difference is very slight or hidden by deceptive obfuscation techniques used by attackers^29^. Furthermore, QDA offers interpretable decision boundaries that reflect the impact of sizable discriminative features, such as uncommon characters in URLs, abrupt packet reordering, or unusual server metadata. Such interpretability helps in understanding how the model distinguishes between the two groups, thereby enhancing trust and transferability to the realm of cybersecurity.

Nevertheless, QDA does have some restrictions. The requirement for each class to have its covariance matrix estimated separately makes the model more sensitive to the dataset size. If there are limited or imbalanced samples, QDA is likely to overfit, particularly in high-dimensional feature spaces. Consequently, this research embeds QDA as part of a hybrid model to deal with these problems, in which (a) the parameters are adjusted with the help of the optimization algorithms and (b) the ensemble strategies provide the system with greater robustness. Such a union not only allows QDA to maintain its power in modeling non-linear class boundaries but also to reap the benefits of stability and generality from boosting methods and bio-inspired optimization.

### Mother Optimization Algorithm (MOA)

MOA is a nature-inspired metaheuristic that draws inspiration from a mother’s caring and nurturing attributes, including guidance, teaching, and molding her children’s character, to facilitate their ever-growing development. On the technical side, MOA models this activity as one that requires both exploration and exploitation to be an efficient search of global optima in complicated problem domains^30^. The MOA algorithm is a model of Mother Nature that changes the phases of operation from “Education” (where the algorithm increases diversity in the candidate solutions population and begins the global search), “Advice” (where the comparison of candidate solutions with stronger ones is conducted) and finally “Upbringing” (local improvement of some solutions by fine-tuning their quality and better exploiting local atmospheres). Each of these three phases enables a different type of interaction with the problem, and collectively they help MOA overcome the pitfalls of other nature-inspired algorithms. The global exploration stage ensures that the algorithm is not trapped in a suboptimal region, while the exploitation stage refines the qualities of high-standard solutions to further enhance their accuracy. Thus, MOA is very effective in complex optimizations with high-dimensional, irregular, and non-linear search spaces.

The article is about the use of MOA as a device to tune classification hyperparameters for the identification of malicious websites^31^. Through adaptive fine-tuning, MOA increases the effectiveness of the learning process as well as the predictive power. Besides, it also enables the proper management of dynamic and adversarial cyber environments, making the defense system an agile and intelligent one against these types of attacks. By selecting the best-performing classifiers, such as LDA, QDA, and CatBoost, MOA enhances the hybrid system’s resilience against attacks. The human-like guidance in the solving process contributes to higher detection rates, scalability, and reliability, making the system more efficient in protecting against malicious websites.

In MOA, each agent represents a hyperparameter vector ${\mathrm{x}}_{i}^{t}$ at iteration *t*. The population evolves through three stages:

**Education (exploration)**:

1 $${\mathrm{x}}_{i}^{{t+1}}={\mathrm{x}}_{i}^{t}+{r_1}\left( {{\mathrm{u}} - {\mathrm{l}}} \right) \odot \mathcal{N}\left( {0,1} \right)$$

where ${r_1} \in \left( {0,1} \right)$, ${\mathrm{u}}$ and ${\mathrm{l}}$ are upper and lower bounds, and $\mathcal{N}\left( {0,1} \right)$ is Gaussian noise.

**Advice (guidance toward elite)**:

2 $${\mathrm{x}}_{i}^{{t+1}}={\mathrm{x}}_{i}^{t}+{r_2}\left( {{\mathrm{x}}_{{best}}^{t} - {\mathrm{x}}_{i}^{t}} \right)$$

where ${\mathrm{x}}_{{best}}^{t}$ denotes the best solution.

**Upbringing (local exploitation)**:

3 $${\mathrm{x}}_{i}^{{t+1}}={\mathrm{x}}_{{best}}^{t}+{r_3}\left( {{\mathrm{x}}_{j}^{t} - {\mathrm{x}}_{k}^{t}} \right)$$

$${\mathrm{with~}}j \ne k,{\mathrm{~and~}}{r_3} \in \left( {0,1} \right).$$

These stages balance global exploration and local exploitation. Boundary control is applied after each update. MOA is employed to optimize classifier hyperparameters by maximizing cross-validation accuracy.

### Osprey Optimization Algorithm (OOA)

The OOA is a population-based metaheuristic that draws inspiration from the distinctive foraging and survival habits of ospreys. The algorithm employs the principle of nature to search for the best solutions in the space, much like ospreys, which are perfect examples of the principle’s validity due to their sharp vision, accuracy, and adaptability^32^. OOA imitates the two main behaviors of ospreys, i.e., finding prey and carrying it to a safe place, which are considered as stages of global exploration and local exploitation, respectively.

- Phase one of the algorithm mimics the osprey’s skill at finding and catching fish in an ever-changing habitat. Each feasible solution traverses the search space by identifying the regions that are most suitable, which is supported by the idea of prey spotting. The mentioned operation leads to a greater chance of local optimum avoidance as the range of the search is extended and the flexibility in complicated landscapes of optimization is enhanced.
- The second stage depicts the behavior of the fish eagle taking the caught prey to a safe place to eat. From a computational perspective, this is reworking and consolidating the best solutions found in a limited area of the search space. Through the process of solution fixation and upgrading via localized search, the algorithm not only accelerates convergence but also gains robustness towards the global optimum.

By combining these actions, OOA is able to function at a level where it can still reap the benefits of both exploration and exploitation, thus making it a perfect fit for solving complex functional problems characterized by irregularities, multiple dimensions, or non-linearity^33^. The organic adjustment maintains that the range of solutions remains varied at the beginning of the process, yet gradually concentrates on improvement as the latter progresses to further stages. OOA has been employed in this research to optimize the hyperparameters of classification models that form the framework of a malicious website detection system. By directing the training process toward more efficient parameter settings, OOA improves model stability, reduces the false alarm rate, and increases detection accuracy. The application of OOA, which is based on its versatile nature in biology, aligns well with the security threat environment, where intruders frequently modify URL formats and network operations to evade detection.

OOA models two behaviors: prey searching (exploration) and prey carrying (exploitation).

**Exploration phase**:

4 $${\mathrm{x}}_{i}^{{t+1}}={\mathrm{x}}_{i}^{t}+\alpha r\left( {{\mathrm{x}}_{{rand}}^{t} - {\mathrm{x}}_{i}^{t}} \right)$$

where ${\mathrm{x}}_{{rand}}^{t}$ is a randomly selected solution, $r \in \left( {0,1} \right)$, and $\alpha$ controls exploration intensity.

**Exploitation phase**:

5 $${\mathrm{x}}_{i}^{{t+1}}={\mathrm{x}}_{i}^{t}+\alpha r\left( {{\mathrm{x}}_{{rand}}^{t} - {\mathrm{x}}_{i}^{t}} \right)$$

where $\beta$ governs convergence speed.

The transition from exploration to exploitation is controlled adaptively over iterations. OOA iteratively updates solutions until termination and selects the hyperparameter vector maximizing validation accuracy.

### Optimizer-enhanced classification framework

This study evaluates the impact of bio-inspired optimization algorithms (MOA and OOA) on the hyperparameter tuning of three classifiers: QDA, LDA, and CatBoost. Each optimizer–classifier combination forms a distinct model variant (e.g., QDOA, QDMA, CAOA, CAMA). The optimization process searches for hyperparameter configurations that maximize validation accuracy within cross-validation. No ensemble learning or architectural fusion is performed. Each model operates independently, and the optimized classifier itself generates final predictions.

The primary contribution of this study is a systematic empirical comparison of bio-inspired optimization algorithms applied to discriminant and gradient-boosted classifiers for malicious URL detection.

### Classification metrics

The strength of the suggested models was measured using standard classification metrics typical for cybersecurity research.

- Accuracy reflects the fraction of correctly categorized websites; thus, it can serve as a general measure of the model’s performance.
- Precision enables determining how many of the websites predicted to be malicious are actually malicious, which is crucial for reducing false alarms on legitimate sites.
- Recall (or sensitivity) defines the extent to which the model can accurately identify harmful web sources, thus ensuring that no threats are missed.
- The F1-score is a feature that combines precision and recall as the harmonic mean, which is a balanced way of evaluating when there is a necessary trade-off between discovering new threats and not producing false positives.
- Matthews Correlation Coefficient (MCC) is a balanced measure for binary classification that considers all four entries of the confusion matrix. It is especially useful when classes are imbalanced.
- Area Under the ROC Curve (AUC) measures the model’s ability to rank positive samples higher than negative ones across all possible classification thresholds.

To sum up, these indicators provide a thorough examination of the detection framework, demonstrating its potential to both recognize threats and accurately identify legitimate websites.

6 $$Accuracy=\frac{{{T_P}+{T_N}}}{{{T_P}+{T_N}+{F_P}+{F_N}}}$$

7 $$Precision=\frac{{{T_P}}}{{{T_P}+{F_P}}}$$

8 $$Recall=\frac{{{T_P}}}{{{T_P}+{F_N}}}$$

9 $$F1\_score{\mathrm{~}}=\frac{{2 \times Recall~ \times ~Precision}}{{Recall+Precision}}$$

10 $${\mathrm{MCC}}=\frac{{TP \times TN - FP \times FN}}{{\sqrt {\left( {TP+FP)\left( {TP+FN} \right)\left( {TN+FP} \right)(TN+FN} \right)} }}$$

## Result

The performance of the proposed classification frameworks was evaluated to assess their effectiveness in detecting malicious URLs in both single and hybrid frameworks. Comparative analyses were conducted against conventional classifiers, including QDA, LDA, and CAT, using multiple metrics, including Accuracy, Precision, Recall, F1-score, and specificity. The selection of MOA and OOA is motivated by their adaptive exploration–exploitation mechanisms and minimal parameterization, which are particularly suitable for optimizing mixed continuous–discrete hyperparameter spaces arising in hybrid learning. Unlike traditional evolutionary optimizers, MOA emphasizes convergence stability through maternal population guidance, while OOA employs dynamic pursuit strategies inspired by osprey hunting behavior, enabling efficient traversal of rugged nonconvex landscapes. Their integration allows coordinated optimization of discriminant and boosting components, yielding robust configurations that are difficult to obtain via grid or random search.

The dataset contains both benign and malicious URLs with moderate class balance. Stratified 5-fold cross-validation was adopted to preserve class proportions during training and testing. No resampling or synthetic balancing techniques were applied. Instead, model evaluation relies on multiple complementary metrics, including precision, recall, F1-score, and specificity, to provide a balanced assessment beyond overall accuracy. This multi-metric evaluation mitigates potential bias arising from unequal class representation.

**Fig. 4:**
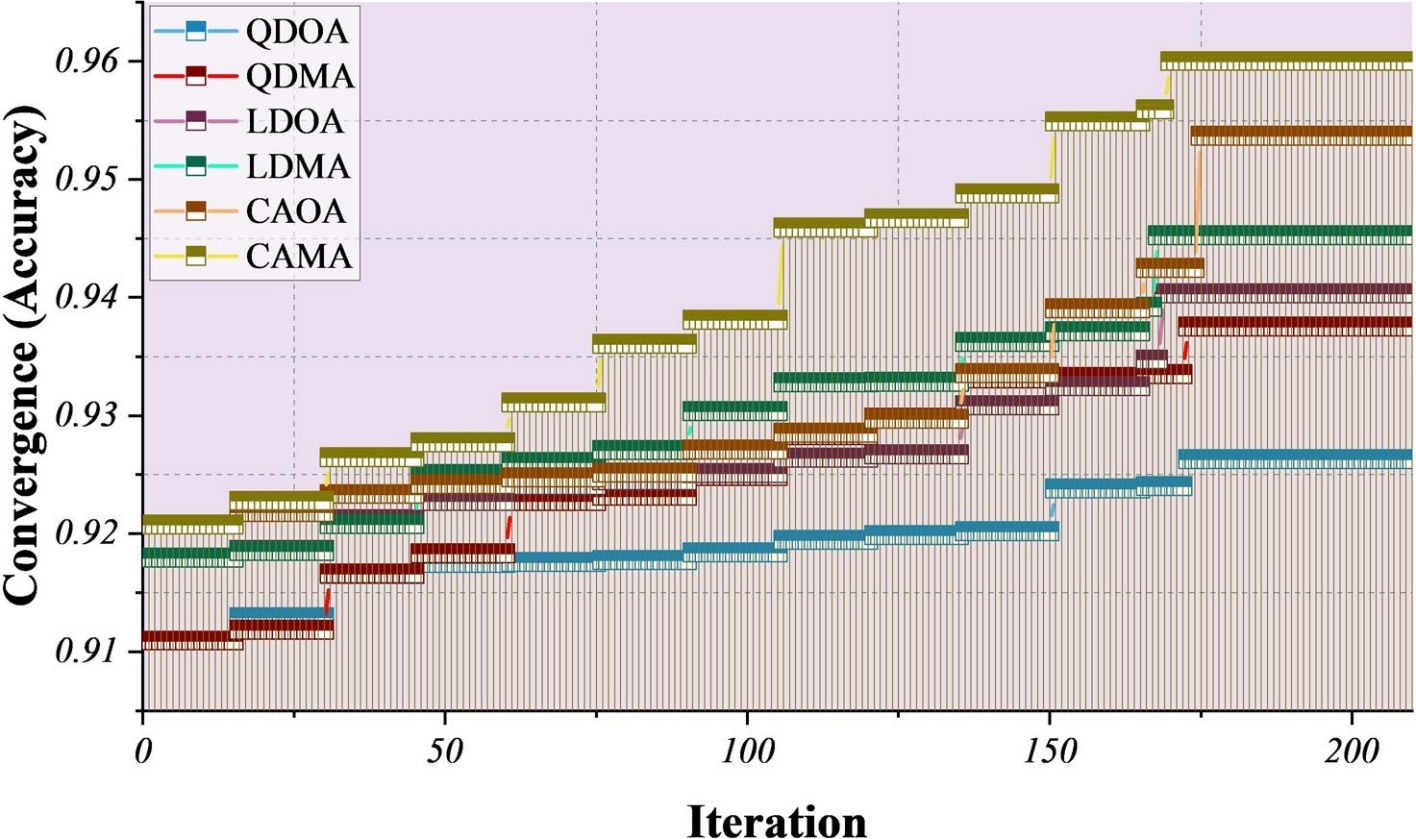
Line-symbol plot for the optimization process’s convergence over iterations, showing continuous improvement in model performance.

The experimental evaluation compared the convergence behavior of the six proposed hybrid models: QDOA, QDMA, LDOA, LDMA, CAOA, and CAMA. Their respective hyperparameters, presented in Table 2, were tuned to ensure a fair and optimized assessment of model performance. For bio-inspired optimization, MOA and OOA were configured with a population size of 50 and a maximum of 210 iterations. These values were selected empirically based on preliminary sensitivity experiments, balancing convergence stability and computational cost. Population sizes below 30 led to premature convergence, whereas values above 50 yielded marginal performance improvements at substantially higher runtime. Similarly, iteration counts beyond 210 yielded negligible gains while increasing optimization overhead. These settings, therefore, represent a practical trade-off between optimization quality and scalability. The experimental evaluation compared the convergence behavior of the six hybrid models: QDOA, QDMA, LDOA, LDMA, CAOA, and CAMA. Their respective hyperparameters, summarized in Table 2, reflect structural diversity across model families: QDOA and QDMA use covariance storage and regularization, LDOA and LDMA rely on solver selection and shrinkage control, and CAOA and CAMA leverage tree depth and learning rate tuning. MOA and OOA jointly optimized these heterogeneous parameters within a unified search process. Figure 4 illustrates convergence trajectories over 210 iterations. All hybrid approaches exhibit monotonic improvement, confirming stable exploration–exploitation behavior. Among the models, CAMA consistently achieves the fastest and highest convergence. Its accelerated progression after approximately 100 iterations highlights the benefit of combining increased tree depth (9) with a higher learning rate (0.17), enabling rapid learning while maintaining stability. CAOA demonstrates similarly strong performance, suggesting that moderate depth (6) and balanced learning rate (0.1) provide an effective compromise between convergence speed and robustness. LDOA and LDMA show intermediate convergence, with LDMA marginally outperforming LDOA due to its higher shrinkage parameter (0.5 versus 0.4), which improves numerical stability under optimization. In contrast, QDOA and QDMA converge more slowly and plateau at lower performance levels. Although covariance storage and regularization enhance generalization, the relatively conservative regularization settings (0.7–0.9) limit adaptability in dynamic search landscapes, resulting in lower optimization efficiency than CatBoost-based hybrids.

**Table 2:** The hyperparameters of the models, along with their assigned values.

Hyperparameter	Hybrid models					
	QDOA	QDMA	LDOA	LDMA	CAOA	CAMA
Store covariance	TRUE	TRUE	-	-	-	-
Reg param	0.7	0.9	-	-	-	-
solver	-	-	lsqr	eigen	-	-
shrinkage	-	-	0.4	0.5	-	-
depth	-	-	-	-	6	9
Learning rate	-	-	-	-	0.1	0.17

To evaluate practical feasibility, runtime and memory consumption were measured for all baseline classifiers and their bio-inspired optimized counterparts. Table 3 summarizes training runtimes and peak memory usage. Baseline discriminant models exhibit negligible training times (LDA: 0.05 s, QDA: 0.08 s), whereas CatBoost requires 0.2 s. Incorporation of bio-inspired optimization increases runtime due to population-based parameter search. Specifically, MOA introduces approximately 2.5–4.0 s overhead, while OOA requires 3.0–4.6 s depending on model complexity. Despite this increase, total training time remains within a few seconds for all configurations. Memory usage follows a similar trend. Plain LDA and QDA consume approximately 5 MB and 20 MB, respectively, while CAT requires 120 MB.

Optimization increases memory demand to 20–25 MB for LDA, 60–70 MB for QDA, and 300–350 MB for CAT, reflecting storage of candidate populations and intermediate model states during optimization. Although MOA and OOA introduce additional computational overhead, this cost is confined to the training phase. Once optimized, inference complexity remains identical to that of the corresponding base models, enabling efficient deployment. These results demonstrate that the framework is scalable for moderate-to-large datasets and suitable for offline training with periodic updates, while maintaining lightweight runtime characteristics during real-time inference.

**Table 3:** Result of the Training time and RAM usage for the developed models.

Model	Runtime (seconds)	RAM usage
QDA	0.08	5 MB
QDMA	3.2	20 MB
QDOA	3.8	25 MB
LDA	0.05	20 MB
LDMA	2.5	60 MB
LDOA	3	70 MB
CAT	0.2	120 MB
CAMA	4	300 MB
CAOA	4.6	350 MB

All experiments were conducted using stratified 5-fold cross-validation to preserve class proportions across folds. A fixed random seed was applied to ensure consistent data partitioning and reproducible results. The performance metrics reported in Table 4 correspond to individual folds, and the final values are obtained by averaging across all folds. For bio-inspired optimization, both MOA and OOA were implemented with a population size of 50 and a convergence criterion of 210 iterations. Optimization was applied exclusively during the training phase. All models were implemented in Python using standard machine learning libraries. Experiments were executed on a workstation equipped with an Intel Core i7 (11th generation) processor and 64 GB RAM.

**Table 4:** The result of the developed K-Fold.

Indicator	Model	Number of K-Folds				
		K1	K2	K3	K4	K5
Accuracy	LDA	0.9148	0.9144	0.9165	0.9179	0.9183
	QDA	0.9056	0.9092	0.9067	0.9089	0.9097
	CAT	0.9248	0.9249	0.9215	0.9228	0.9254

Table 5 presents the evaluation metrics for both single- and hybrid-models during training and testing. Across all metrics, including Accuracy, Precision, Recall, F1-score, and specificity, the hybrid approaches consistently outperformed the single models, underscoring the effectiveness of combining diverse learning strategies. In the training phase, single models such as QDA, LDA, and CAT achieved competitive results, with CAT delivering the strongest performance among them (Accuracy = 0.9207, F1_score = 0.9301, specificity = 0.9394). However, when compared with hybrid models, their predictive ability was noticeably lower. Hybrid models demonstrated progressive improvements: QDOA and QDMA surpassed their single-model counterparts, while LDOA and LDMA further improved precision and F1-score. LDMA achieved an accuracy of 0.9453 and an F1-score of 0.9500, supported by high specificity (0.9496). The strongest results were obtained by CAOA and CAMA, with CAMA attaining the highest training accuracy (0.9600) and balanced performance across all metrics (Precision = 0.9694, Recall = 0.9600, specificity = 0.9611). These results indicate that deeper architectures and carefully tuned learning rates (as seen in CAMA) can substantially enhance model expressiveness during training.

**Fig. 5:**
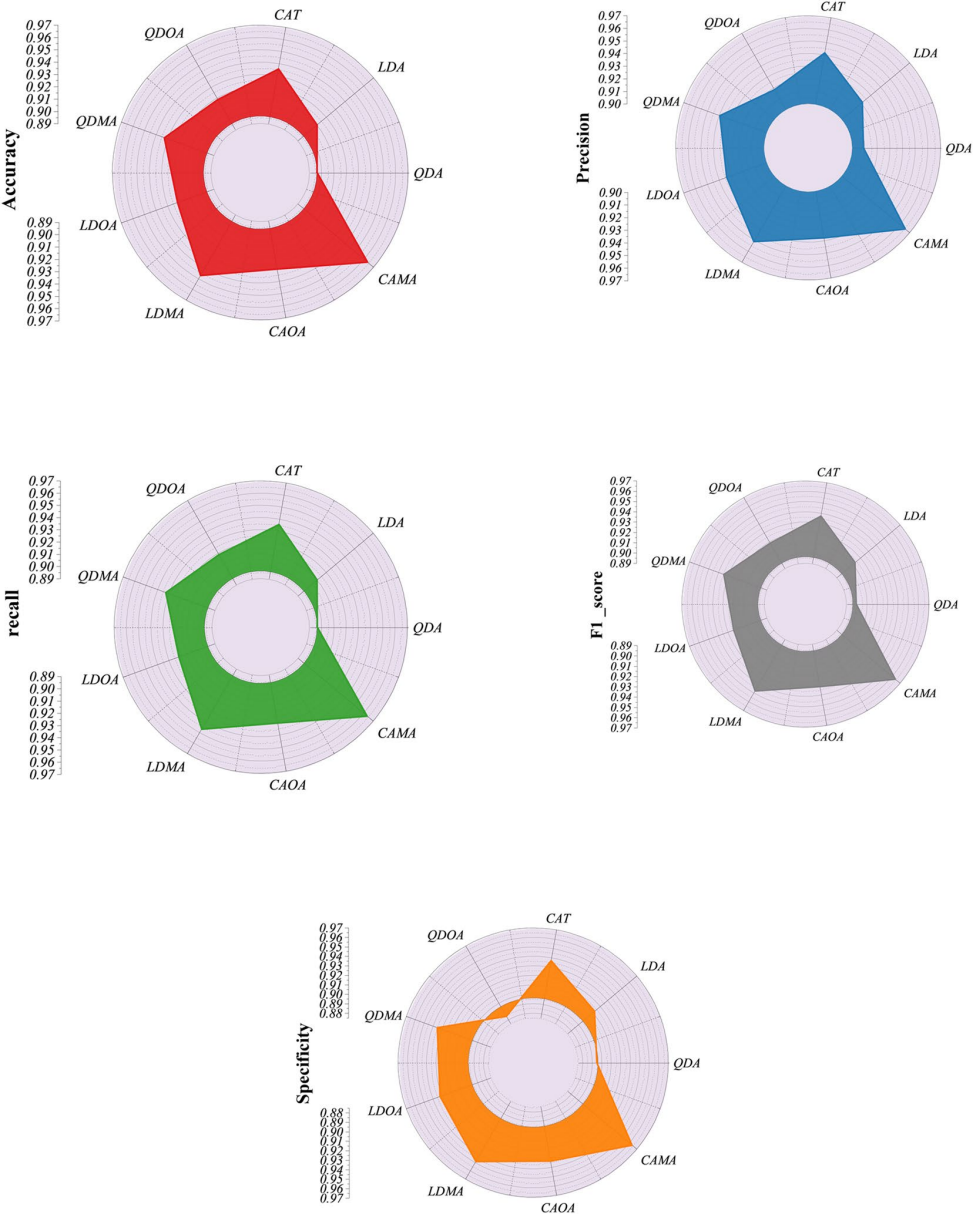
Radial plot for the performance metrics of the developed models.

The superiority of hybrid models was even more evident in the testing phase. While single models performed adequately, with CAT again leading (Accuracy = 0.9354, F1_score = 0.9370), they were consistently outperformed by hybrids. Among the hybrid group, LDMA achieved high generalization capability (Accuracy = 0.9466, F1_score = 0.9477, specificity = 0.9484), confirming its robustness beyond the training set. The CAMA model demonstrated the best overall test performance, with Accuracy = 0.9635, Precision = 0.9654, Recall = 0.9635, F1_score = 0.9640, and specificity = 0.9636. The balance across all metrics highlights its ability to reduce both false positives and false negatives, which is critical for reliable deployment in practical scenarios. Figure 5 compares the developed models using the specified metrics.

**Table 5:** Evaluation metrics were employed to measure the predictive accuracy and overall performance of the models.

Phase	Category	Models	Metrics						
			Accuracy	Precision	Recall	F1 _score	Specificity	MCC	AUC
Train	Single	*QDA*	0.9109	0.9432	0.9109	0.9210	0.9003	0.633	0.900
		*LDA*	0.9179	0.9490	0.9179	0.9273	0.9210	0.666	0.921
		*CAT*	0.9207	0.9535	0.9207	0.9301	0.9394	0.688	0.939
	Hybrid	*QDOA*	0.9263	0.9511	0.9263	0.9339	0.9223	0.685	0.922
		*QDMA*	0.9375	0.9561	0.9375	0.9431	0.9318	0.720	0.932
		*LDOA*	0.9404	0.9566	0.9404	0.9453	0.9300	0.726	0.930
		*LDMA*	0.9453	0.9618	0.9453	0.9500	0.9496	0.755	0.950
		*CAOA*	0.9537	0.9646	0.9537	0.9569	0.9475	0.778	0.948
		*CAMA*	0.9600	0.9694	0.9600	0.9626	0.9611	0.808	0.961
Test	Single	*QDA*	0.8961	0.9088	0.8961	0.8995	0.8945	0.738	0.895
		*LDA*	0.9101	0.9212	0.9101	0.9130	0.9120	0.773	0.912
		*CAT*	0.9354	0.9415	0.9354	0.9370	0.9369	0.833	0.937
	Hybrid	*QDOA*	0.9185	0.9182	0.9185	0.9184	0.8840	0.771	0.884
		*QDMA*	0.9326	0.9395	0.9326	0.9343	0.9351	0.826	0.935
		*LDOA*	0.9213	0.9336	0.9213	0.9241	0.9319	0.806	0.932
		*LDMA*	0.9466	0.9511	0.9466	0.9477	0.9484	0.860	0.948
		*CAOA*	0.9298	0.9376	0.9298	0.9317	0.9332	0.820	0.933
		*CAMA*	0.9635	0.9654	0.9635	0.9640	0.9636	0.902	0.964

**Fig. 6:**
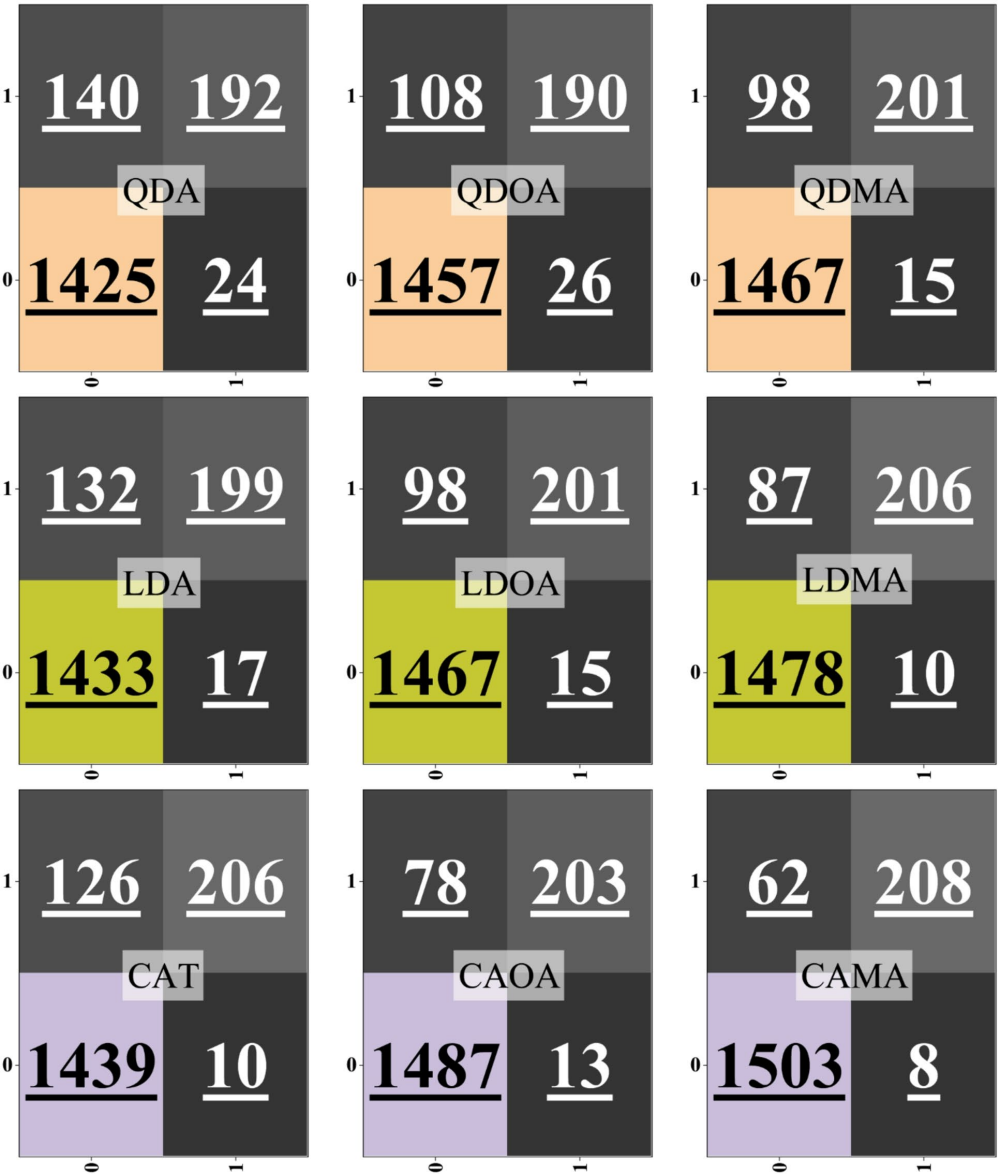
Confusion matrix plot for the classification performance of the model across different classes.

Figure 6 illustrates the confusion matrices for single (QDA, LDA, CAT) and hybrid (QDOA, QDMA, LDOA, LDMA, CAOA, CAMA) models, providing deeper insight into classification behavior across the two classes. The diagonal elements represent correct predictions, while the off-diagonal values correspond to misclassifications. Among the single models, CAT demonstrated the strongest performance, with 1,439 true negatives and only 10 false positives, indicating a strong ability to minimize Type I errors. LDA also performed relatively well, with 1,433 true negatives and 17 false positives, whereas QDA lagged, misclassifying a larger portion of positive samples (192 false negatives), resulting in lower recall. The hybrid models consistently achieved higher correct classification counts than their single-model counterparts. QDOA and QDMA improved upon QDA, reducing both false positives and false negatives, with QDMA achieving a particularly balanced trade-off (1,467 true negatives and 201 true positives). Similarly, LDOA and LDMA outperformed LDA, with LDMA further reducing misclassification rates (1,478 true negatives and 206 true positives), reflecting the robustness indicated by its strong test metrics in Table 5. CAOA and CAMA delivered the most accurate predictions across both classes. CAOA achieved 1,487 true negatives with only 13 false positives, while CAMA provided the best overall performance, with 1,503 true negatives and 208 true positives, accompanied by just 8 false positives and 62 false negatives. This minimal error distribution demonstrates that CAMA not only excels in overall accuracy but also achieves a balanced reduction in both false-negative and false-positive rates.

Table 6 reports the Precision, Recall, and F1_score of all models under two distinct conditions (0 = malicious and 1 = benign URLs). The analysis reveals consistent trends across both groups, with hybrid models obtaining more desirable performance compared to single models, particularly in Condition 1, which appears more challenging given the generally lower precision and F1-score values. In Condition 0, all models achieved high performance, with precision values exceeding 0.98 and F1-scores above 0.94. Among single models, LDA and CAT performed strongly, though QDA lagged slightly behind with an F1-score of 0.9456. Hybrid models further improved performance, with LDMA (F1_score = 0.9682), CAOA (F1_score = 0.9703), and especially CAMA (F1_score = 0.9772) achieving the highest balance across precision and recall. These results highlight the advantage of hybridization in extracting richer feature representations, even under easier conditions. In Condition 1, the differences between models became more pronounced. Single models such as QDA (F1_score = 0.7007) and LDA (F1_score = 0.7276) showed limited capacity to maintain balanced precision and recall, reflecting weaker adaptability in more complex settings. CAT showed improvement (F1_score = 0.7518), but remained below hybrid alternatives. Among hybrids, QDOA and QDMA offered moderate improvements with F1-scores of 0.7393 and 0.7806, respectively. LDMA performed notably better (F1_score = 0.8094), demonstrating its robustness across conditions. CAOA achieved a strong balance (Precision = 0.7224, Recall = 0.9398, F1_score = 0.8169), while CAMA achieved the best performance overall (Precision = 0.7704, Recall = 0.9630, F1_score = 0.8560). Overall, the results suggest that hybrid models generalize more effectively across varying conditions. While performance differences were marginal in Condition 0, hybrid models, particularly LDMA, CAOA, and CAMA, exhibited significantly stronger resilience in Condition 1. CAMA’s consistently high Precision, Recall, and F1_score across both conditions further confirm its superiority as the most effective and reliable model in this comparative study.

**Table 6:** Comparative assessment of model performance under varying conditions.

Model	Condition	Metrics		
		Precision	Recall	F1 _score
QDA	0	0.9834	0.9105	0.9456
	1	0.5783	0.8889	0.7007
QDOA	0	0.9825	0.9310	0.9560
	1	0.6376	0.8796	0.7393
QDMA	0	0.9899	0.9374	0.9629
	1	0.6722	0.9306	0.7806
LDA	0	0.9883	0.9157	0.9506
	1	0.6012	0.9213	0.7276
LDOA	0	0.9899	0.9374	0.9629
	1	0.6722	0.9306	0.7806
LDMA	0	0.9933	0.9444	0.9682
	1	0.7031	0.9537	0.8094
CAT	0	0.9931	0.9195	0.9549
	1	0.6205	0.9537	0.7518
CAOA	0	0.9913	0.9502	0.9703
	1	0.7224	0.9398	0.8169
CAMA	0	0.9947	0.9604	0.9772
	1	0.7704	0.9630	0.8560

**Fig. 7:**
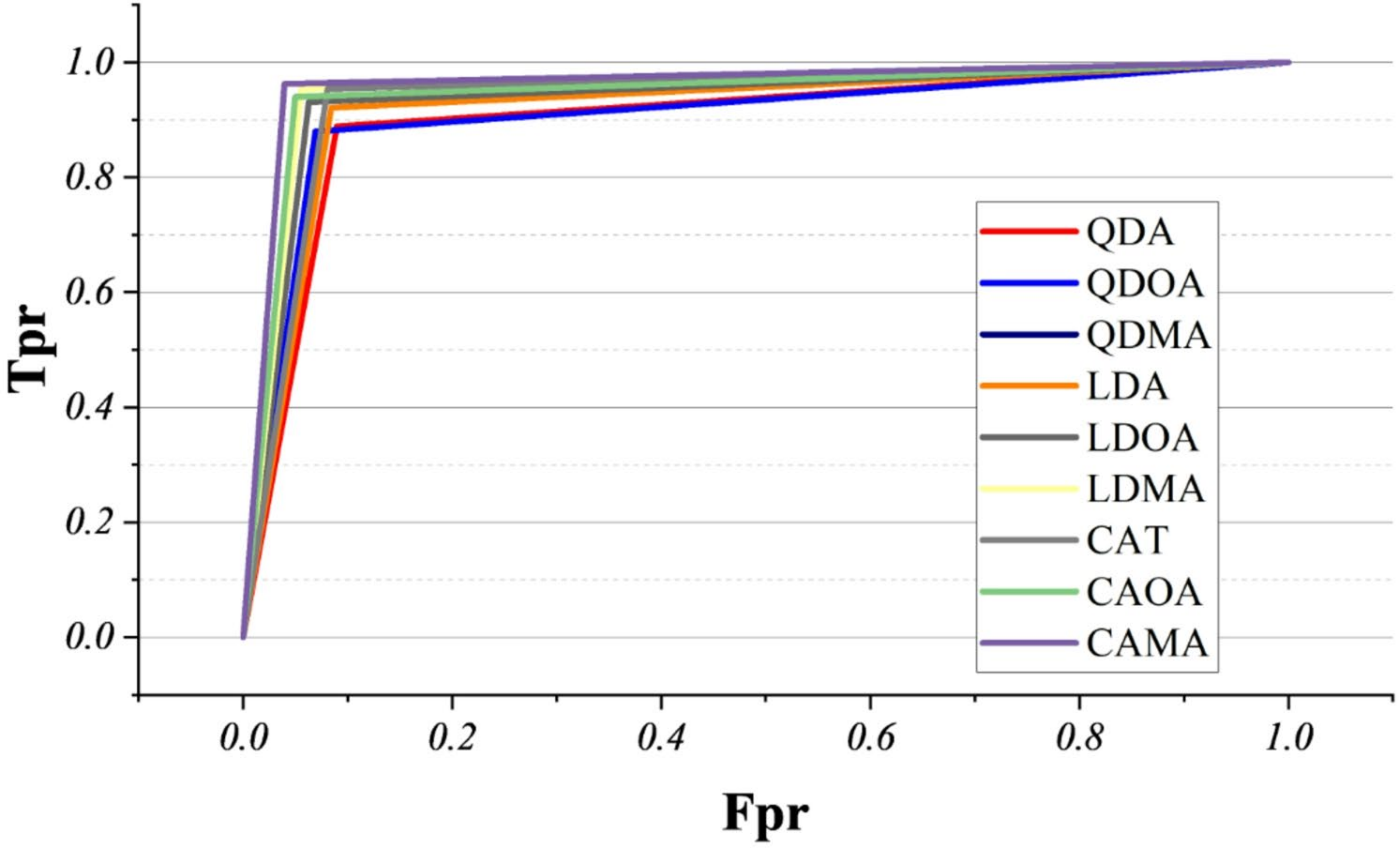
ROC curve for the balance between sensitivity and false positive rate.

The ROC curves, in Fig. 7, illustrate the trade-off between sensitivity (true positive rate) and specificity (1 − false positive rate) across the evaluated models. All classifiers demonstrated strong discriminative ability, with curves concentrated near the upper-left corner of the plot, indicating high sensitivity at low false positive rates. Among them, CAMA, CAOA, and LDMA exhibited the steepest ascent and largest enclosed area, suggesting superior classification performance. In contrast, traditional QDA and QDOA showed slightly lower sensitivity at comparable false-positive rates, reflecting a relatively weaker generalization capacity. Overall, the adaptive variants (e.g., CAMA, CAOA, LDMA) consistently outperformed the baseline linear and quadratic models, highlighting their robustness in achieving near-optimal separation between classes.

To assess whether performance improvements introduced by bio-inspired optimization are statistically significant, the Wilcoxon signed-rank test was applied to compare baseline models with their optimized counterparts across cross-validation folds. This nonparametric test evaluates paired differences in model accuracy without assuming normality. Table 7 reports the Wilcoxon statistics and corresponding p-values for each model configuration. All optimized variants exhibit statistically significant improvements (*p* < 0.05), confirming that MOA- and OOA-based tuning yields consistent gains over unoptimized baselines. This analysis complements the Kruskal–Wallis feature-level assessment by providing statistical validation at the model level.

**Table 7:** Result of the Wilcoxon test.

Models	Parameter	
	Statistical	*P* value
QDA	1980	1.33E-19
QDOA	1755	1.40E-12
QDMA	855	5.81E-15
LDA	1275	4.46E-21
LDOA	855	5.81E-15
LDMA	490	5.36E-15
CAT	685	2.60E-23
CAOA	598	9.50E-12
CAMA	284	1.09E-10

**Fig. 8:**
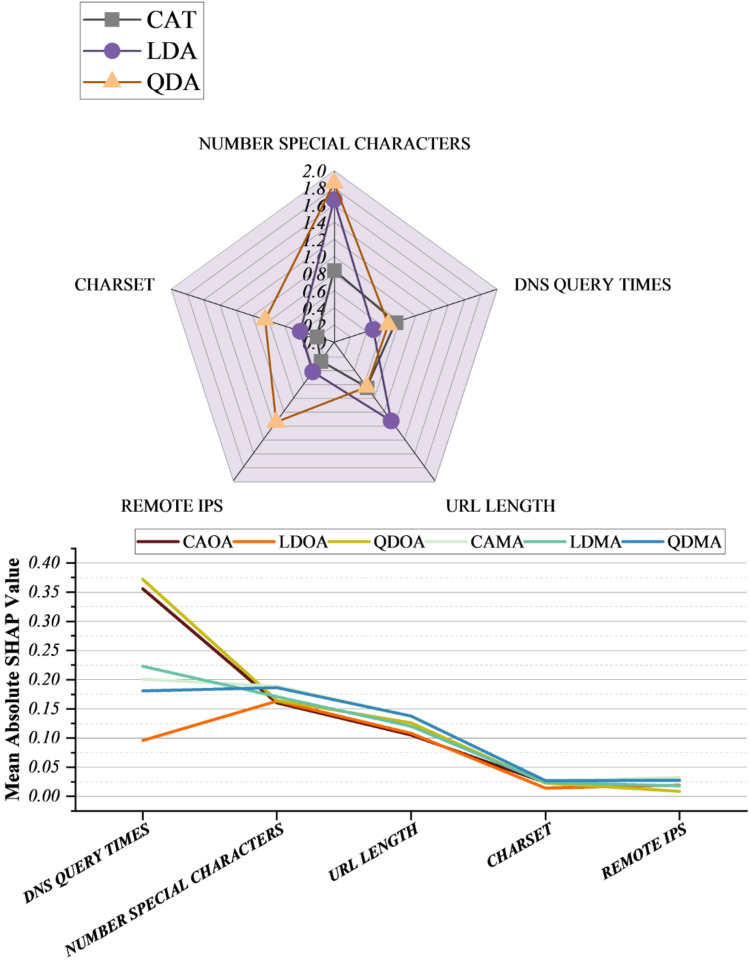
SHAP sensitivity for the impact of each feature on model predictions.

Figure 8 presents the SHAP-based feature sensitivity for all hybrid models (CAOA, CAMA, LDOA, LDMA, QDOA, and QDMA), highlighting the relative contribution of URL- and network-level attributes after optimization with MOA and OOA. Across all hybrid configurations, DNS Query Times and Number of Special Characters emerge as the most influential predictors, followed by URL Length, while Charset and Remote IPs exhibit comparatively lower but non-negligible contributions. The prominence of Number of Special Characters in the LDA- and QDA-based hybrids reflects its practical relevance for detecting obfuscated or deceptive URLs, which commonly contain characters such as @, %, or ? to imitate legitimate addresses or conceal malicious intent. DNS Query Times, consistently ranked as the most impactful feature in the CAT-based hybrids, capture network-level irregularities that may arise from repeated redirections, unstable hosting, or command-and-control communication patterns, thereby supporting early identification of malicious infrastructure. URL Length also shows substantial influence across multiple hybrids, aligning with well-known obfuscation strategies in which excessively long URLs are used to hide payloads or redirect users to harmful resources. Remote IP usage, particularly evident in the QDA-based hybrids, indicates attempts to bypass domain-based filtering by directly referencing IP addresses, a technique frequently employed in phishing campaigns and malware hosting. Charset-related anomalies, emphasized in the CAT hybrids, further suggest the presence of encoded or injected content designed to evade signature-based detection. Overall, the SHAP analysis demonstrates that the hybrid models leverage a complementary set of structural URL features and network indicators, providing interpretable evidence of how optimization-enhanced classifiers distinguish benign from malicious websites. This interpretability supports a layered defense strategy, enabling security teams to prioritize monitoring based on feature importance and facilitating automated URL filtering and adaptive threat detection in real-world cybersecurity environments.

## Discussion

### Limitations and future work

Although the proposed hybrid computational intelligence framework demonstrates competitive performance in malicious URL detection, several limitations should be acknowledged. First, the relatively modest dataset size (1,781 samples) constrains the ability to generalize findings to broader, real-world internet traffic, which often exhibits greater diversity and higher volume. While the full dataset was used without down-sampling, limited sample diversity may affect robustness against rare or emerging attack patterns. Second, although integrating discriminative models with bio-inspired optimization enhances accuracy, the computational cost of meta-heuristic search and ensemble inference may pose challenges for real-time or resource-constrained deployment. Finally, the framework currently focuses on structured features derived from URL syntax and network attributes; richer semantic content derived from page text or behavior logs remains unexplored.

Future work can extend this study in several promising directions. Recent advances in modular, composable large language models (LLMs) offer a potential pathway to deeper semantic analysis and flexible reasoning in cybersecurity tasks. By incorporating modular LLM components capable of contextual interpretation of URLs and content, the framework could achieve greater adaptability to novel obfuscation strategies and evolving threat semantics^34^. Additionally, loss functions tailored for imbalanced learning, such as focal loss, have demonstrated the capacity to prioritize hard-to-classify instances while maintaining computational efficiency. Integrating focal loss into the boosting or ensemble stages may further improve performance on challenging samples without significant overhead^35^.

Beyond methodological enhancements, future research should also investigate scalability to internet-scale datasets, the incorporation of online or continual learning mechanisms to adapt to emergent threats, and cross-domain evaluation across diverse network environments. Collectively, these extensions can strengthen both the practical applicability and theoretical foundations of hybrid computational intelligence in cybersecurity. Although SHAP enhances model transparency, explanations may be influenced by feature correlations, leading to shared attribution among dependent variables. In addition, SHAP provides associative rather than causal interpretations. The present study evaluates SHAP on static test folds; future work will explore temporal explainability under concept drift and integrate decorrelation strategies to refine feature attribution further.

The proposed framework is evaluated on a single static dataset and does not incorporate temporal splitting or external benchmark datasets. Consequently, robustness to evolving threats and long-term generalization cannot be conclusively established. While the framework leverages behavioral and structural features that are less sensitive to signature drift, future work will explicitly address concept drift through time-aware training (e.g., training on older URLs and testing on newer samples) and validation on multiple independent datasets. These extensions will enable more rigorous assessment of deployment readiness in dynamic cybersecurity environments.

## Conclusion

This study introduced and systematically evaluated a suite of hybrid classification frameworks, including Quadratic Discriminant Analysis (QDA) with Osprey Optimization Algorithm (OOA) (QDOA), QDA with mother optimization algorithm (MOA) (QDMA), Linear Discriminant Analysis (LDA) with OOA (LDOA), LDA with MOA (LDMA), CATBoost with OOA (CAOA), and CATBoost and MOA (CAMA), by integrating discriminant analysis and hybrid learning with optimization strategies. These hybrid models were benchmarked against conventional classifiers, containing QDA, LDA, and CAT, to assess their ability to enhance predictive performance in malicious URL detection. Experimental findings demonstrated that the hybrid frameworks consistently improved learning stability, convergence behavior, and overall classification accuracy. Among the models, CAMA demonstrated the highest performance, achieving superior results across key metrics, including accuracy, precision, recall, F1-score, and specificity, in both the training and testing phases. The model maintained a robust balance between sensitivity and specificity, confirming its capacity to generalize effectively across diverse conditions. LDMA and CAOA also delivered strong performance, significantly surpassing traditional classifiers and demonstrating resilience to class imbalance, a frequent challenge in cybersecurity applications. Model interpretability was further explored using SHAP-based sensitivity analysis. Results indicated that the hybrid models leveraged the most informative features more effectively than baseline models, producing clearer and more discriminative decision boundaries. Performance improvements were not solely the result of increased model complexity but also reflected a more efficient utilization of the underlying feature space. Feature contributions varied across models, emphasizing the ability of hybrid frameworks to capture subtle patterns in malicious URL data. In general, these results highlight the benefits of combining discriminant analysis with adaptive learning methods to create interpretable, robust, and scalable classifiers. The hybrid architectures, mainly CAMA, offer an exciting possibility to enhance the trustworthiness and openness of phishing and malicious URL detection systems. By marrying high predictive accuracy with the explainability of the decision process, these models lay a practical foundation for cybersecurity solutions in volatile digital environments, where performance, generalization, and trustworthiness are imperative. The findings validate the potential of hybridization between classical and adaptive methods as a tool capable of effectively handling the complexity of the evolving threats scenario, thus contributing to a resilient approach to digital security without compromising the ability to track the importance of features. The present study is validated on a dataset of 1,781 samples. While sufficient for methodological evaluation, future work should examine scalability on substantially larger and more diverse datasets to confirm robustness in true internet-scale environments.

## Data Availability

The dataset will be provided upon reasonable request. The source of the dataset is provided in Sect.  2.1.
